# Structural basis for the assembly of the DNA polymerase holoenzyme from a monkeypox virus variant

**DOI:** 10.1126/sciadv.adg2331

**Published:** 2023-04-19

**Authors:** Yaning Li, Yaping Shen, Ziwei Hu, Renhong Yan

**Affiliations:** ^1^ Center for Infectious Disease Research, Westlake Laboratory of Life Sciences and Biomedicine, Key Laboratory of Structural Biology of Zhejiang Province, School of Life Sciences, Westlake University, Hangzhou, Zhejiang Province 310024, China.; ^2^ Beijing Advanced Innovation Center for Structural Biology, Tsinghua-Peking Joint Center for Life Sciences, School of Life Sciences, Tsinghua University, Beijing 100084, China.; ^3^ Department of Biochemistry, Key University Laboratory of Metabolism and Health of Guangdong, School of Medicine, Southern University of Science and Technology, Shenzhen, Guangdong Province 518055, China.

## Abstract

The ongoing global pandemic caused by a variant of the monkeypox (or mpox) virus (MPXV) has prompted widespread concern. The MPXV DNA polymerase holoenzyme, consisting of F8, A22, and E4, is vital for replicating the viral genome and represents a crucial target for the development of antiviral drugs. However, the assembly and working mechanism for the DNA polymerase holoenzyme of MPXV remains elusive. Here, we present the cryo–electron microscopy (cryo-EM) structure of the DNA polymerase holoenzyme at an overall resolution of 3.5 Å. Unexpectedly, the holoenzyme is assembled as a dimer of heterotrimers, of which the extra interface between the thumb domain of F8 and A22 shows a clash between A22 and substrate DNA, suggesting an autoinhibition state. Addition of exogenous double-stranded DNA shifts the hexamer into trimer exposing DNA binding sites, potentially representing a more active state. Our findings provide crucial steps toward developing targeted antiviral therapies for MPXV and related viruses.

## Teaser

Cryo-EM structure of the MPXV DNA polymerase holoenzyme is revealed, providing clues into drug design against poxviruses.

## INTRODUCTION

A recent outbreak of a variant of the monkeypox (or mpox) virus (MPXV) has rapidly spread to over 110 countries, with over 80,000 confirmed cases (as of 26 November 2022) since early May of 2022 (https://cdc.gov/poxvirus/monkeypox/response/2022/world-map.html) (*1*). The World Health Organization (WHO) has declared this global monkeypox pandemic to be a public health emergency of international concern and is calling for research into vaccines, therapeutics, and other tools to fight this virus (*2*).

MPXV is a member of Poxviridae, a family of enveloped, double-stranded DNA (dsDNA) viruses that also includes the variola virus, the causative agent of smallpox that has claimed millions of lives in the 20th century (*3*–*5*). Phylogenetic analysis has revealed two main clades: West Africa (WA) and Congo Basin (CB) (*6*, *7*). While the fatality rate for the CB clade is typically 1 to 5%, patients with WA clade cases are rarely fatal in non-immunocompromised patients (*8*). Genome sequencing shows that the current MPXV outbreak originated from a 2018 WA strain but contains 50 extra mutations (*9*).

MPXV is a contagious zoonotic virus, first isolated from monkeys, that can be transmitted to humans through close contact with infected animals (*6*). It can also infect a variety of mammals such as rope squirrels, Gambian pouched rats, African hedgehogs, chimpanzees, and more (*7*, *10*). Human-to-human spread typically occurs through direct contact with infectious rash, sores, and scabs (*11*). In addition, many confirmed cases in this outbreak have been traced to sexual transmission (*12*).

During productive infection, members of the Orthopoxvirus family, such as MPXV, replicate their genome in virus-derived perinuclear sites of the infected cell using a set of virus-encoded proteins (*13*–*15*). Similar to vaccinia virus (VACV), another well-studied member of Poxviridae, the DNA polymerase holoenzyme of MPXV encoded by the viral genome contains three proteins: the uracil-DNA glycosylase E4, the processivity factor A22, and the DNA polymerase F8 belonging to the B-family polymerases, which correspond to D4, A20, and E9 in VACV, respectively (*16*–*18*). A22 works as a bridge for connecting F8 and E4, among which E4 is crucial for the association of viral DNA templates with enzymes to process efficiently (*19*–*23*).

**Fig. 1.**
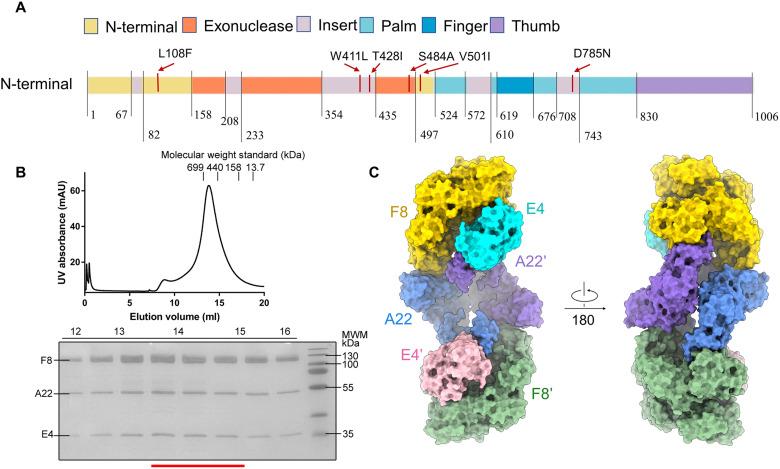
Biochemical characteristics of DNA replication machine of MPXV. (**A**) Domain organization of F8L. Mutations in the 2022 WA strain are marked by red lines. (**B**) Representative SEC purification of the F8-A22-E4 complex. SDS-PAGE was visualized by Coomassie blue staining, and fractions for cryo-EM analysis were marked by red line. (**C**) Overall surface presentation of domain-colored cryo-EM structures of DNA replication machine of monkeypox. F8, A22, and E4 in protomer A are colored gold, blue, and cyan, respectively, and the other protomer is colored green, purple, and pink.

Many attempts targeted to the DNA polymerase holoenzyme of Poxviridae are developed, such as the acyclic cytosine phosphonate analog cidofovir (CDV) and the CDV-derived prodrug brincidofovir (also known as CMX001), but DNA polymerase mutant variant conferred resistance to these drugs (*24*–*27*). There are six extra mutations (L108F, W411L, T428I, S484A, V501I, and D785N) located on F8 DNA polymerase from the 2022 WA strain of MPXV compared with previous strains (Fig. 1A) (*9*, *28*). Notably, the expression of DNA polymerase E9 of VACV is strictly controlled during the infection process (*29*, *30*). The levels of E9 mRNA transcription and translation peaked at about 2 to 3.5 hours after infection and then decline, becoming barely detectable by 5 to 6.5 hours after infection, probably for transition into late gene expression (*29*). However, the molecular mechanism for the regulation of the DNA polymerase activity during the infection process remains unclear.

Despite the insights gained from VACV structural research, including the crystal structures of DNA polymerase E9 alone, the processivity factor A20 (1 to 50 amino acids)/D4 complex, C-terminal domain of A20 (304 to 426 amino acids), and low-resolution model of E9/A20/D4 (*17*, *19*, *22*, *23*), there remains very limited knowledge concerning the precise assembly and action mechanism for the holoenzyme of Poxviridae. Here, we present the cryo–electron microscopy (cryo-EM) structure of the DNA replication holoenzyme F8/A22/E4 from the 2022 WA strain (GenBank ID: ON563414.3) at an overall resolution of 3.5 Å. Unexpectedly, unlike any other previously reported B-family polymerase, the holoenzyme complex is assembled as a dimer of heterotrimers. To the best of our knowledge, this is the first time that the hexameric assembly of B-family polymerase has been revealed. Structural analysis illustrated that in the hexameric form of the DNA polymerase holoenzyme from MPXV, the DNA polymerase F8 directly interacts with the uracil-DNA glycosylase E4, in contrast to the previous low-resolution models from VACV, which assumed that the complex is elongated in shape and the DNA polymerase and uracil-DNA glycosylase are separated (*20*). The extra interface between the thumb domain of F8 and the middle Neck domain of A22′ might preclude the binding of DNA substrate; this could hint at an inactive conformation. When supplied with an exogenous dsDNA, the hexameric form of the holoenzyme changed into a trimeric form, notably exposing the DNA binding site of the thumb domain. Besides, the high-resolution structure of F8 provides a framework for further analysis of each domain and the emerged mutations, which could lead to improved understanding of the availability of drugs targeting the holoenzyme.

## RESULTS

### Structure determination of F8/A22/E4 holoenzyme

To determine its structure, the open reading frames of *F8L/A22R/E4R* from the 2022 West African strain were coexpressed and purified in human embryonic kidney 293 freestyle (HEK293F) cells through a combination of tandem affinity resin and size exclusion chromatography (SEC). The purified complex showed high homogeneity, as it eluted in a single mono-disperse peak (Fig. 1B). Details of cryo-sample preparation, data acquisition, and structural determination are provided in Materials and Methods and sections of the Supplementary Materials (figs. S1 to S5 and table S1). A 3D reconstruction was obtained at a resolution of 3.5 Å from 391,085 selected particles, revealing the dimer of heterotrimers’ architecture (Fig. 1C).

**Fig. 2.**
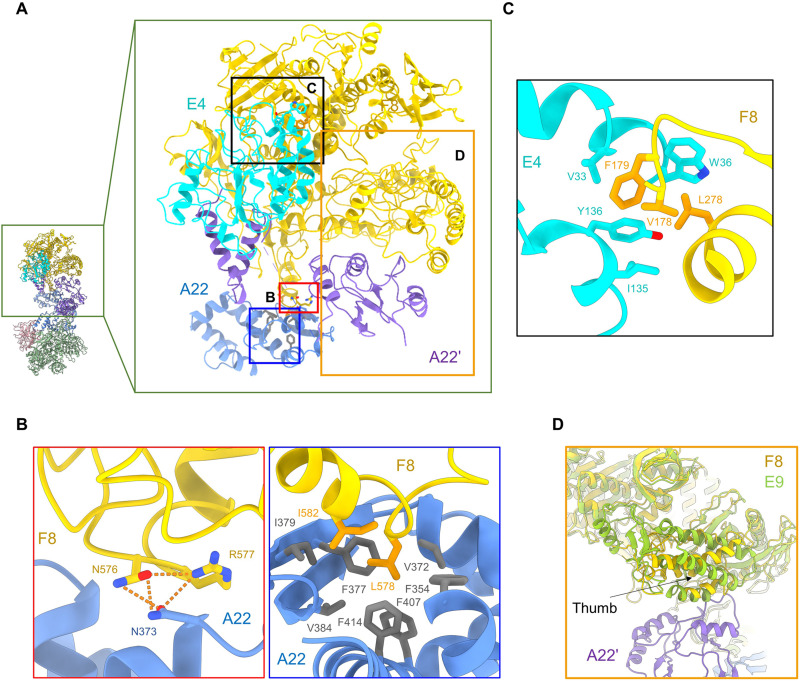
The multiple interaction interfaces in the F8-A22-E4 hexameric complex. (**A**) Overall structure of the F8-A22-E4 complex. Interaction interfaces between F8 and A22 in one protomer are lined out by red and blue boxes, which are shown in (**B**). The interacted residues in F8 and E4 are lined out by black box and shown in (**C**). (**D**) Structural comparison with E9 DNA polymerase from VACV (PDB ID: 5N2E) shows that the thumb domain is regulated by the interaction of A22′. F8, E4, A22, and A22′ are colored gold, cyan, blue, and purple, respectively. E9 is colored yellow green.

The high-resolution structure allowed for precise model building. The structure of the F8 polymerase showed an open conformation, with 927 of its 1006 side chains assigned, including the N-terminal, exonuclease, palm, fingers, thumb domains, and five “poxvirus-specific” inserts (insert 0 to insert 4) (Figs. 1A and 2A) (*20*). The emerged mutations are clearly mapped on the structure (fig. S6A). The overall architecture of F8 is similar to that of VACV, with a root mean square deviation of 4.40 Å for 929 Cα, with the thumb domain being notably shifted due to its interaction with the Neck domain of A22′ (Fig. 2D) (*17*). The full-length structure of A22 has been resolved, connecting F8 and E4, and the overall structure of E4 is similar to its homologous protein, D4, in VACV (*20*).

### Multiple interfaces of holoenzyme

The structure of A22 is divided into three parts: the N-terminal domain, the middle Neck domain, and the C-terminal domain. The linker (46 to 101 amino acids) between the N-terminal domain and the Neck domain is invisible when high resolution is reached, but the low-contour threshold could help identify the direction of this chain (fig. S6B).

For clarity, two protomers are referred to as protomer A: F8/A22/E4 and protomer B: F8′/A22′/E4′. The overall architecture exhibits a central symmetry, creating an extra interface: the Neck domain of A22 and the thumb domain of F8′ (Fig. 2). However, the density of the interface between the Neck domain of A22′ and the thumb domain of F8 is not good enough to support detailed model building, due to the flexibility of the interface (Fig. 2D and fig. S4D). Overall, A22 plays a bridge role in binding the N-terminal domain of A22 to E4′ and C-terminal domain of A22 interaction with the poxvirus-specific insert 3 region of F8.

The overall architecture exhibits a central symmetry, which is related by two twofold axes of rotation and linked by interactions between the A22′ Neck domain and the F8′ thumb domain. The focused refinement is applied on protomer A, which improves the resolution up to 3.3 Å, enabling detailed analysis (Fig. 2). The interface between F8 and C-terminal domain of A22 is mainly mediated by the short helices of poxvirus-specific insert 3 region that inserts into the hydrophobic pocket of A22. The extensive hydrophobic network contains Leu^578^ and Ile^582^ of F8 and Phe^354^, Val^372^, Phe^377^, Ile^379^, Val^384^, Phe^407^, and Phe^414^ of A22. Meanwhile, Asn^576^ and Arg^577^ of F8 are hydrogen-bonded (H-bonded) with Asn^373^ of A22 (Fig. 2B). In addition, the interface between F8 and E4 is mainly mediated by the hydrophobic interactions involved in Val^178^, Phe^179^, and Leu^278^ of F8 and Val^33^, Trp^36^, Ile^135^, and Tyr^136^ of E4 (Fig. 2C). Compared with E9, the homolog polymerase in VACV, the thumb domain is notably shifted probably due to the interaction with the Neck domain of A22′ (Fig. 2D).

### Distinct organization pattern

**Fig. 3.**
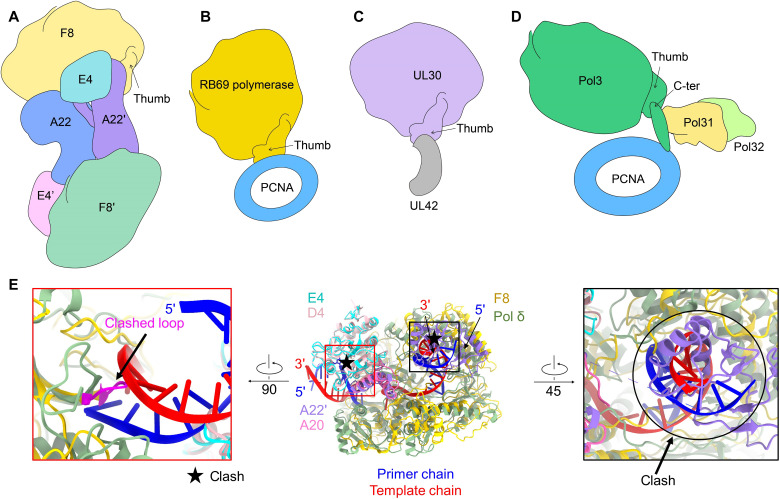
The models of different DNA replication machines. (**A**) Model of DNA polymerase holoenzyme from MPXV. (**B**) Archaeal and phage DNA polymerase. The model was drawn according to polymerase structure (PDB ID: 1CLQ) and PCNA structure (PDB ID: 1B8H). (**C**) HSV DNA polymerase. The model was drawn according to UL42 structure (PDB ID: 1DML) and UL30 structure (PDB ID: 2GV9). (**D**) *Saccharomyces cerevisiae* polymerase. The model was drawn according to a complex structure (PDB ID:7KC0). (**E**) Structural comparison with DNA polymerase delta of yeast (PDB ID: 3IAY) and D4-A20 complex of VACV (PDB ID: 4YIG) shows that there would be a clash between DNA and F8 or A22′. F8, A22, A22′, and E4 are colored gold, blue, purple, and cyan, respectively. D4 and A20 of VACV are colored pink and magenta. DNA polymerase delta of yeast is colored dark sea green. The template and primer chains of DNA are colored red and blue.

Previous sequence analysis of the VACV polymerase has revealed the homology to the replicative B-family of PALM polymerases (Pol α and Pol δ) encoded by mammalian cells and a variety of DNA viruses such as herpes simplex virus (HSV) and bacteriophage RB69 (*17*, *31*). The B-family polymerases usually accommodate some cofactors for processivity (*32*). However, the organization pattern of different members of B-family polymerases varies widely (Fig. 3, A to D). The bacteriophage RB69 acquires the trimeric sliding clamp factor proliferating cell nuclear antigen (PCNA) through the C-terminal thumb domain of polymerase (Fig. 3B) (*33*). Similarly, the PCNA-related processivity factor (UL42) of HSV also binds to the C-terminal thumb domain of the polymerase (UL30) (Fig. 3C) (*34*). Eukaryotic polymerase δ acquires more processivity factors such as Pol31 and Pol32 during the replication process, while still retaining the ability to bind PCNA through the elongated C-terminal domain (Fig. 3D) (*35*). Compared to other B-family polymerases, the replication machine in MPXV is characterized by the presence of a base-excision enzyme, the uracil-DNA glycosylase E4, and a dimeric organization pattern (Fig. 3, A to D).

The unexpected organization pattern of the DNA polymerase holoenzyme of MPXV raises the concern of the working mechanism. In VACV, the uracil glycosylase activity of E4 has been shown to be active in the context of the polymerase holoenzyme and plays a role like the sliding clamp, as evidenced by the in vitro replication assay that could generate abasic sites in a uracil-containing oligonucleotide and viral DNA replication and repair could be coupled (*36*). However, there is an obvious clash between the substrate uracil-containing DNA and F8 in the solved holoenzyme structure of MPXV, suggesting an inactive form of E4 in this structure (Fig. 3E, left). To be noticed, when aligning F8 with yeast DNA polymerase δ, the thumb domain of F8 is engaged by the Neck domain of A22′ and also shows a clash between A22′ and substrate dsDNA, suggesting an inhibitory effect of A22 on the elongation of DNA polymerase in this structure (Fig. 3E, right). Together, these findings suggest that the hexameric form of DNA polymerase holoenzyme from MPXV might represent as an inactive conformation.

### Two different states of holoenzyme reveal the underlying working mechanism

**Fig. 4.**
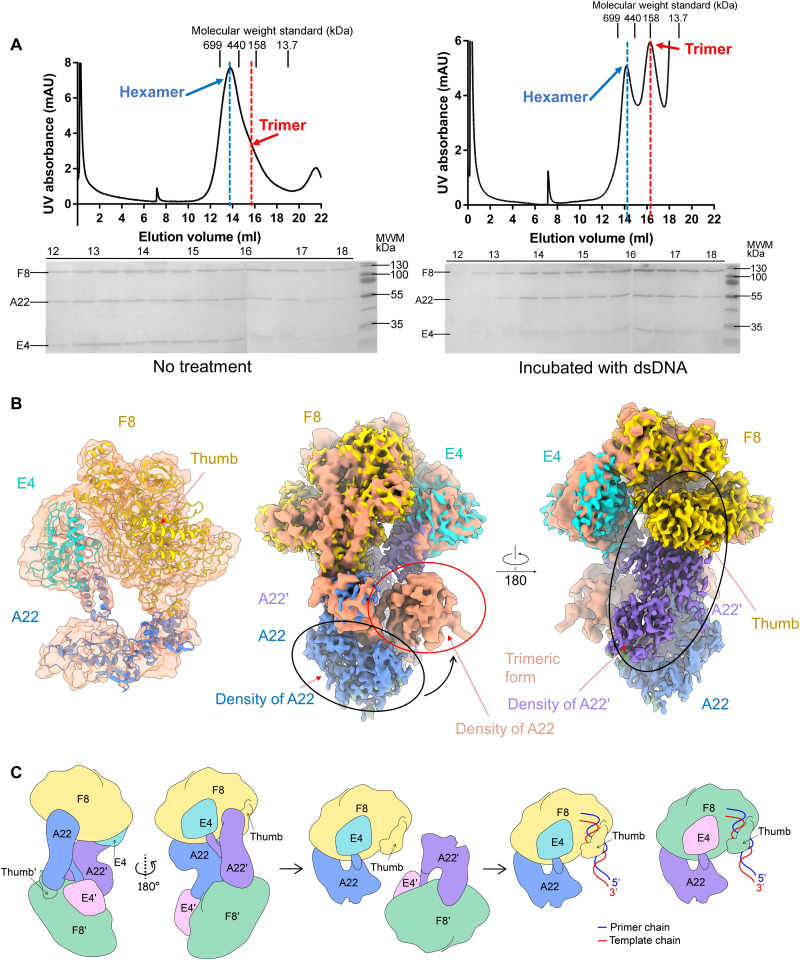
The putative working model of the F8-A22-E4 complex. (**A**) The oligomeric state shift of the F8-A22-E4 complex from hexamer to trimer can be induced by incubation with dsDNA. The peaks of hexamer and trimer are marked by blue line and red line, respectively, on SEC analysis. The major peak of the F8-A22-E4 complex shifts backward when incubated with dsDNA. (**B**) Left: Map and model of trimeric form complex. The map of trimeric form complex is colored light salmon. The model styled cartoon and F8, A22, and E4 are colored gold, blue, and cyan, respectively. Middle and right: Map comparison of the trimeric and hexameric form complex shows that A22 undergoes marked conformational change during oligomer state shift. (**C**) Proposed model for autoinhibition, activation, and the DNA replication mechanism catalyzed by the F8-A22-E4 complex.

To explore the molecular mechanism of DNA replication by MPXV, we tried to incubate the dsDNA substrate (see Materials and Methods) with the hexameric complex form. To our surprise, the addition of the exogenous dsDNA leads to the increase of a latter peak on gel filtration and it still contains the bands of F8/A22/E4 on SDS–polyacrylamide gel electrophoresis (SDS-PAGE), which might represent another state of the holoenzyme (Fig. 4A). We then collected the latter peak fractions and prepared the cryo-EM sample. Structural determination reveals the structure of trimeric form with F8/A22/E4 at the resolution of 3.1 Å (Fig. 4B, figs. S3 and S5, and table S1). However, we could not get the DNA substrate-bound structure when using both peak fractions.

The trimeric structure of the F8/A22/E4 complex might exhibit as a more active state due to the loss of the interface between the thumb domain of F8 and A22′, thus releasing the dsDNA binding site of F8 (Fig. 4B). The transition from hexameric form to trimeric form requires a marked movement of the N-terminal domain and the Neck domain of A22, in which the dissociation of A22′ could lead to the replacement of the interface of A22′-E4 into A22-E4 (Fig. 4B and fig. S6B).

## DISCUSSION

The replication process of poxviruses is a complex and tightly regulated process. The peak of mRNA and protein levels of E9 in VACV is 2 to 3.5 hours after infection and declines to be undetectable by 5 to 6.5 hours, aiming for transition into late gene expression state (*29*). Besides, BAF (barrier to autointegration factor) dimers can bind tightly to dsDNA and occlude transcription and replication of VACV; however, B1-mediated phosphorylation of BAF prevents this inhibitory effect (*37*, *38*). Many studies have shown that the polymerase alone of poxvirus is intrinsically distributive but the processivity factor could render it highly processive, suggesting the critical role of A22 and E4 (*16*, *39*). The molecular mechanism that regulates the activity of the DNA replication machine of poxvirus remains elusive. The replication process is studied over time using VACV as the prototype poxvirus. The primer- and template-dependent DNA synthesis requires a short double-stranded sequence to begin synthesis. The rolling hairpin replication mechanism suggests that the free ends of linear DNA genomes fold over to produce self-priming hairpin loops (*40*). However, the identification of DNA helicase-primase (D5 in VACV) suggests a replication fork mechanism (*41*). The conditional lethal D5 mutants show a fast stop DNA replication phenotype, suggesting a critical role of D5 in the DNA replication process (*42*).

Here, we found the two different states of DNA replication holoenzyme from MPXV, which could be induced by the dsDNA to transition an inactive hexameric form into a more active trimeric form. We proposed a possible working model for the DNA replication holoenzyme by the F8/A22/E4 complex based on our findings (Fig. 4C). In the proposed model, the autoinhibitory hexameric form of holoenzyme could not exert the replication function because the DNA binding site of F8 and E4 is not exposed. Once the dsDNA substrate or another factor binds to the holoenzyme, it can trigger a conformational change into a trimeric complex, releasing the DNA binding site of the thumb domain. This triggers a replication process that transforms the inhibition conformation of F8 into an active conformation to bind the template-primer strands. Structural comparison among F8 in trimeric form/hexameric form holoenzyme complex and E9 alone [Protein Data Bank (PDB) ID:5N2E] reveals little change in exonuclease domain. As the exonuclease activity in F8 in trimeric form was confirmed (*43*), we suggest that F8 in hexameric form and E9 alone could also completely degrade the primer DNA in an adenosine 5′-triphosphate (ATP)–independent manner (fig. S7A).

**Fig. 5.**
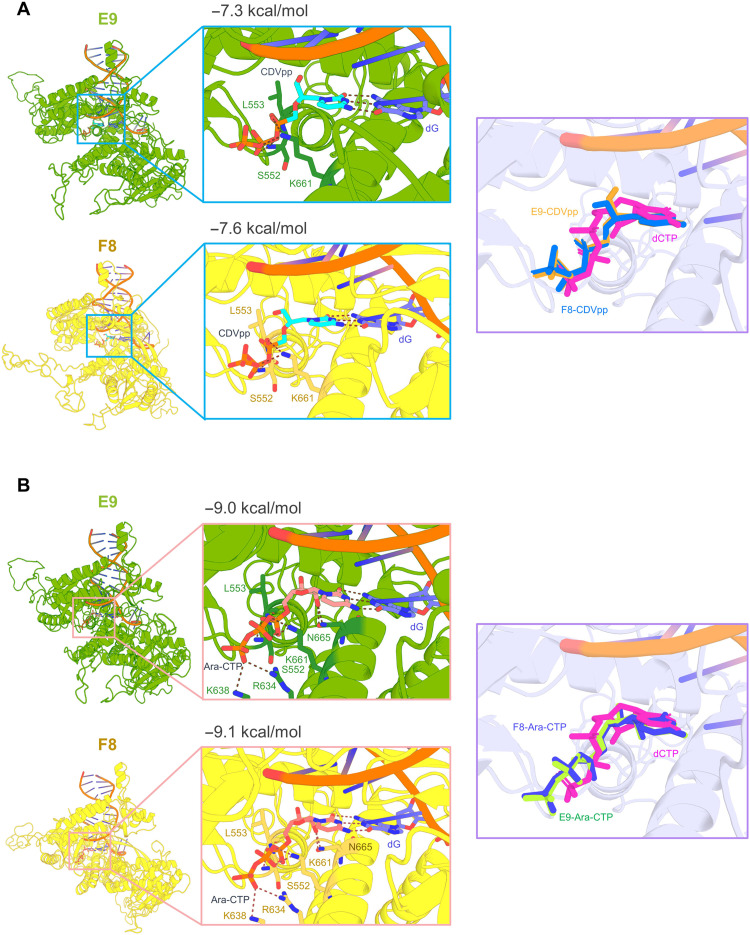
Analysis of drug inhibitors on E9 and F8. (**A**) Molecular docking of CDVpp binding mode in E9 and F8, respectively. The interaction residues are shown as sticks. E9 and F8 are colored split pea and yellow, respectively. The structure on the right is the comparative analysis of dCTP (light magenta) and CDVpp in E9 and F8, respectively. (**B**) Molecular docking of Ara-CTP in E9 and F8. The interaction residues are shown as sticks. The alignment analysis on the right is also the comparative analysis of dCTP (light magenta) and Ara-CTP in E9 and F8, respectively.

We also wonder about the role of CDV and CDV-derived prodrug CMX001 against MPXV (*44*, *45*). It is reported that CDV could be metabolized into the triphosphate form [cidofovir diphosphate (CDVpp)] to be served as competitive inhibitor of deoxycytidine triphosphate (dCTP) binding to inhibit a growing DNA strand and destabilize the dsDNA of virus (*27*, *46*, *47*). The previously reported structures of B-family polymerases in complex with DNA substrate, with or without an incoming nucleotide mimicking elongation mode, indicate that large domain movements happen upon DNA binding, leading to a closure of the structure compared to the open conformation (*48*). To study the effect of CDV on viral DNA polymerase, molecular docking analysis was carried out to estimate the CDVpp binding between E9 of VACV and F8 of MPXV. We used the yeast polymerase δ structure with bound template and complementary DNA (PDB ID: 3IAY) to generate the elongation mode of F8 and E9 by SWISS-MODEL (fig. S7, B and C) (*48*, *49*). From our docking results, CDVpp exhibited considerable binding affinity to closed conformation of E9 and F8, −7.3 and −7.6 kcal/mol, respectively. We found that CDVpp incorporated into the DNA chain opposite the dG in a state strikingly similar to the incoming dCTP. CDVpp enters into the active cavity of F8 or E9 via conventional hydrogen bonds to the main chain of Ser^552^ and Leu^553^, and side chain of Lys^661^, which are also critical to stabilize the incoming natural dCTP (Fig. 5A). These results suggested that CDV and its analog have high effectiveness in the treatment of MPXV infection. Another dCTP competitor of DNA synthesis, cytarabine triphosphate (Ara-CTP), which is the active metabolite of cytarabine, also shows a considerable binding affinity with E9 (−9.0 kcal/mol) and F8 (−9.1 kcal/mol), indicating a putative therapeutic candidate (*50*). Ara-CTP is capable of acting as direct chain terminators, which is involved in the hydrogen bonding network with the main chain of Leu^553^ and Ser^552^and the side chain of three positively charged Lys^638^, Lys^661^, Arg^634^, and Asn^665^ located at the vicinity of the dCTP binding pocket (Fig. 5B). The triphosphate tails of both agents interact with the active site, in a manner analogous to that previously reported with deoxynucleotide triphosphates (dNTPs) in the structure of the yeast polymerase δ structure. Moreover, hydrogen bonding interactions are also observed between the dG and either CDVpp or Ara-CTP to sustain their binding stability, further precluding the binding of incoming dNTPs.

The remodeling structure of F8 and our reported structures provide an important clue into the putative effect of emerged mutations of MPXV (fig. S8). For an overall view, L108F mutation is close to the template chain of dsDNA and may strengthen the hydrophobic interaction with bases in template chain. W411L and T428I are both located on the insert 2 region of F8, which may interact with the finger domain upon dsDNA binding as does “polymerase-associated domain” in Y-family polymerases (*28*).

One remaining major question is why we were unable to capture a DNA-bound structure at first? To be honest, this problem troubled us for a long time. Recently, we introduced exonuclease-deficient mutations (E166A and E168A), which are involved in Mg^2+^ ion binding at exonuclease domain and designed a DNA duplex with the primer containing a dideoxycytosine at its 3′ end after literature research. Fortunately, these two optimization strategies have helped us capture the DNA-bound structure. During the revision of this manuscript, another group reported the structures of MPXV DNA polymerases bound with the DNA substrate (*43*), which also certified these strategies and provided more insight into the working mechanism of the replication machine.

In summary, the high-resolution structure of the MPXV DNA polymerase holoenzyme reveals the mode of processivity factor binding and provides an understanding of putative working mechanisms, although the dynamic conformational change of the holoenzyme still requires further structural and functional research to obtain more functional states. The identification of the interfaces of F8/E4 and F8/A22 might facilitate the design of antiviral drugs that disrupt these interactions.

## MATERIALS AND METHODS

### Protein expression and purification

The full-length *F8L* of MPXV (MPXV 2022 West African strain, GenBank: ON563414.3) was cloned into the pCAG vector (Invitrogen) with a C-terminal 10× His tag. The full-length *E4R* and *A22R* (MPXV 2022 West African strain, GenBank: ON563414.3) were cloned into the pCAG vector (Invitrogen) with an N-terminal FLAG tag. All the plasmids used to transfect cells were prepared by the GoldHi EndoFree Plasmid Maxi Kit (CWBIO).

The recombinant protein was overexpressed using the HEK293F mammalian cells at 37°C under 5% CO_2_ in a Multitron-Pro shaker (Infors, 130 rpm). When the cell density reached 2.0 × 10^6^ cells/ml, the plasmid was transiently transfected into the cells. To transfect 1 liter of cell culture, about 1.5 mg of the plasmid was premixed with 3 mg of polyethylenimines (Polysciences) in 50 ml of fresh medium for 15 min before adding to cell culture. Cells were collected by centrifugation at 4000*g* for 10 min after 60 hours of transfection and resuspended in a buffer containing 25 mM Hepes (pH 7.5), 150 mM NaCl, and mixture of three protease inhibitors, aprotinin (1.3 μg/ml; AMRESCO), pepstatin (0.7 μg/ml; AMRESCO), and leupeptin (5 μg/ml; AMRESCO).

For F8/A22/E4 complex purification, cells were lysed by sonication and cell debris was removed by centrifugation at 18,700*g* for 45 min. The supernatant was loaded onto anti-FLAG M2 affinity resin (Sigma-Aldrich). The resin was washed with the wash buffer containing 25 mM Hepes (pH 7.5) and 150 mM NaCl, followed by protein elution with wash buffer plus FLAG peptide (0.2 mg/ml). Then, elution of anti-FLAG M2 affinity resin was further purified with Ni-NTA affinity resin (Qiagen). Wash buffer and elution buffer of nickel resin was wash buffer mentioned above plus 10 and 300 mM imidazole. Then, the protein complex was subjected to SEC (Superose 6 Increase 10/300 GL, GE Healthcare) in buffer containing 25 mM Hepes (pH 7.5) and 150 mM NaCl. The peak fractions were collected and concentrated for EM analysis.

### DNA-induced conformational change assay

The DNA duplex used for the assay is the 12/16 primer template from the Pol δ-DNA complex (PDB ID:3IAY), containing the 12-nucleotide (nt) oligonucleotide primer (5′-ATCCTCCCCTAC-3′) annealed to the 16-nt template (5′-TAAGGTAGGGGAGGAT-3′) (*48*).

Fresh F8/A22/E4 complex from peak fractions of SEC is incubated with the DNA duplex at a molar ratio of about 1:3 plus 2 mM MgCl_2_ for 1 hour. Protein was incubated with 2 mM MgCl_2_, and the same volume buffer only without the DNA duplex is used as negative control. Then, the mixture was subjected to SEC (Superose 6 Increase 10/300 GL, GE Healthcare) in buffer containing 25 mM Hepes (pH 7.5) and 150 mM NaCl.

### Cryo-EM sample preparation and data acquisition

For complex in hexameric form, the fresh protein complex was concentrated to ~3 mg/ml and applied to the grids. For complex in trimeric form, the latter peak from the DNA-induced conformational change assay was collected and concentrated to ~3 mg/ml. Aliquots (3.3 μl) of the protein were placed on glow-discharged holey carbon grids (Quantifoil Au R1.2/1.3). The grids were blotted for 3.0 or 3.5 s and flash-frozen in liquid ethane cooled by liquid nitrogen with Vitrobot (Mark IV, Thermo Fisher Scientific). The prepared grids were transferred to a Titan Krios operating at 300 kV equipped with a Gatan K3 detector and a GIF Quantum energy filter. Movie stacks were automatically collected using EPU software (Thermo Fisher Scientific), with a slit width of 20 eV on the energy filter and a defocus range from −1.4 to −1.8 μm in superresolution mode at a nominal magnification of ×81,000. Each stack was exposed for 2.56 s with an exposure time of 0.08 s per frame, resulting in a total of 32 frames per stack. The total dose rate was approximately 50 e^−^/Å^2^ for each stack. The stacks were motion-corrected with MotionCor2 (*51*) and binned twofold, resulting in a pixel size of 1.095 or 1.072 Å/pixel. Meanwhile, dose weighting was performed (*52*). The defocus values were estimated with Gctf (*53*).

### Data processing

The cryo-EM structure of the complex was solved in Relion 3.0.6 and cryoSPARC. Particles were automatically picked using Relion 3.0.6 (*54*) from manually selected micrographs. After two-dimensional (2D) classification with Relion, good particles were selected and subjected to 2D classification, ab initio reconstruction, and multiple cycles of heterogeneous refinement without symmetry using cryoSPARC (*55*). The good particles were selected and subjected to local CTF refinement with C1 symmetry, nonuniform refinement, resulting in the 3D reconstruction for the whole structures. To further improve the resolution, the particles of nonuniform refinement were subjected to 3D classification and focused refinement with Relion.

The resolution was estimated with the gold standard Fourier shell correlation 0.143 criterion (*56*) with high-resolution noise substitution (*57*). Refer to the Supplementary Materials, figs. S1 to S5, and table S1 for details of data collection and processing.

### Model building and structure refinement

Predicted models of F8, E4, and A22 were first obtained using AlphaFold 2.1 (*58*), which was further manually adjusted based on the cryo-EM map with Coot (*59*). Each residue was manually checked with the chemical properties taken into consideration during model building. Several segments, whose corresponding densities were invisible, were not modeled. Structural refinement was performed in Phenix (*60*) with secondary structure and geometry restraints to prevent overfitting. To monitor the potential overfitting, the model was refined against one of the two independent half maps from the gold standard 3D refinement approach. Then, the refined model was tested against the other map. Statistics associated with data collection, 3D reconstruction, and model building were summarized in table S1.

### Molecular docking

Docking simulation with the software AutoDock Vina (version 1.2.0) was used to investigate interaction of proteins and ligands, according to the following procedures: (i) preparation of template structures of remodeled E9 and F8. We used the yeast polymerase δ structure with bound template and complementary DNA (PDB ID: 3IAY) to generate the elongation mode of F8 and E9 implemented in SWISS-MODEL (https://swissmodel.expasy.org) (*49*). The models were processed by removing existing ligands, while the missing polar hydrogen was added using AutoDockTool software. Subsequently, it was saved into a dockable PDBQT format for the following virtual work. (ii) Docking ligand preparation. The structure data file format of small molecules (CDVpp and Ara-CTP) was obtained from the PubChem website (https://pubchem.ncbi.nlm.nih.gov) and minimized energy using MM2 algorithm. The PDBQT chemical formats of CDVpp and Ara-CTP were created by AutoDock Tool software. (iii) Set docking grid box. The AutoDockTool software was used to build up docking grid box parameter files of E9 and F8, respectively, based on the active pocket where reference molecule (dCTP) was located. Grid Box (22 Å × 30 Å × 36 Å) centered at (6.679, 0.395, 34.289) Å, for the E9, grid box (30 Å × 30 Å × 30 Å) centered at (6.509, 0.395, 34.521) Å, for the F8. (iv) Molecular docking was performed by AutoDock Vina, and the final results were processed with PyMOL (version 1.4.1).

## Notes

View/request a protocol for this paper from *Bio-protocol*.
