# Supplementary material for: Structural basis for the assembly of the DNA polymerase holoenzyme from a monkeypox virus variant
Source: Sci Adv. 2023 Apr 19;9(16):eadg2331. doi: 10.1126/sciadv.adg2331 (PMC10115419; doi:10.1126/sciadv.adg2331)
Supplement: Supplementary file 1 — Figs. S1 to S8 Table S1 [file sciadv.adg2331_sm.pdf]

Science Advances
AAAS

Supplementary Materials for

Structural basis for the assembly of the DNA polymerase holoenzyme from a monkeypox virus variant

Yaning Li *et al.*

Corresponding author: Renhong Yan, yanrh@sustech.edu.cn

Sci. Adv. **9**, eadg2331 (2023)
DOI: 10.1126/sciadv.adg2331

This PDF file includes:

Figs. S1 to S8
Table S1

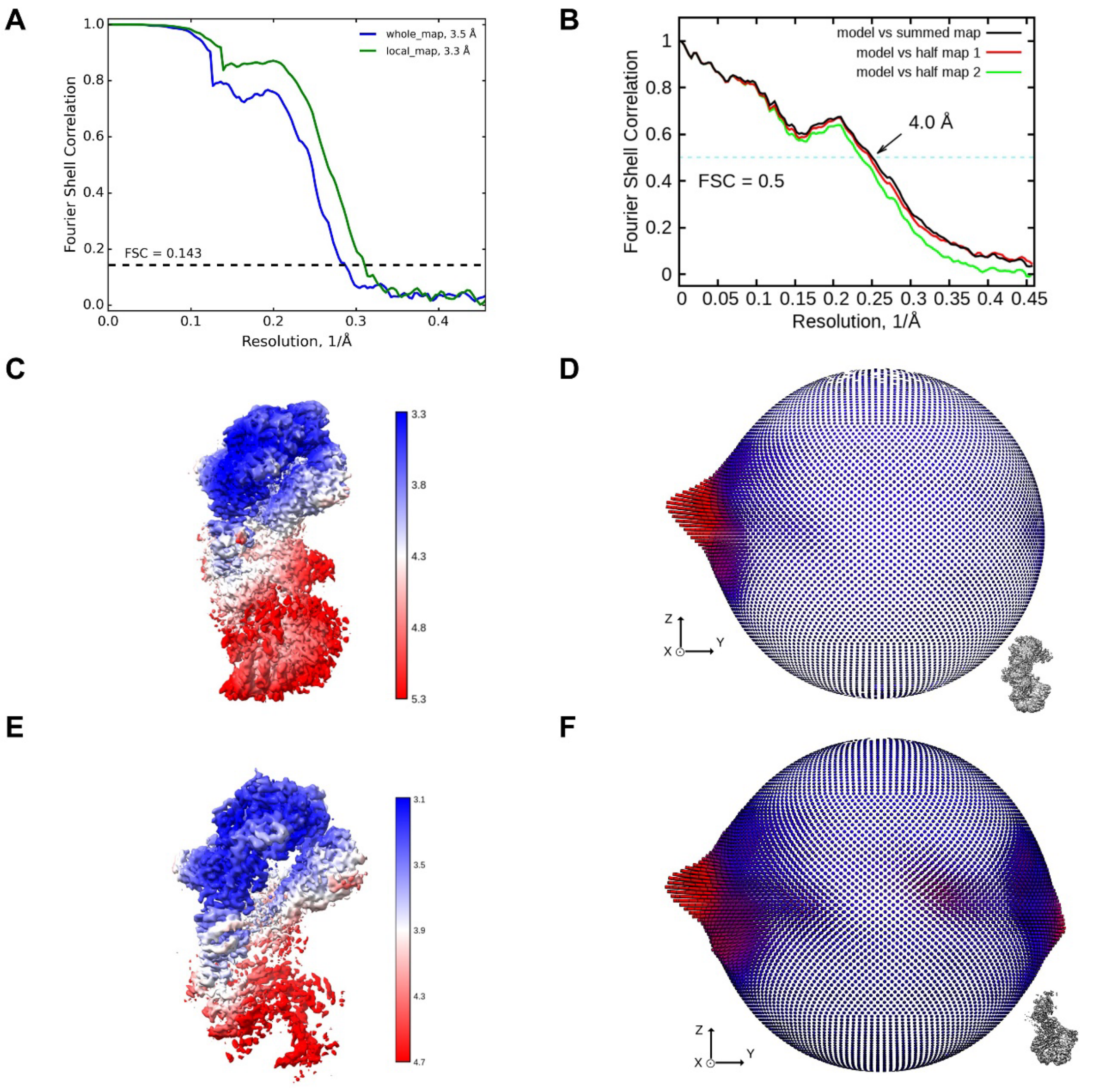

Fig. S1

Cryo-EM analysis of F8/A22/E4 complex in hexameric form.

(A) FSC curve. The resolution was estimated with the gold-standard Fourier shell correlation 0.143 criterion with high-resolution noise substitution. **(B)** FSC curve of the refined model versus the overall structure that it is refined against (black); of the model refined against the first half map versus the same map (red); and of the model refined against the first half map versus the second half map (green). The small difference between the red and green curves indicates that the refinement of the atomic coordinates did not suffer from overfitting. **(C)** Local resolution map for the 3D reconstruction of the overall structure. **(D)** Euler angle distribution in the final 3D reconstruction of

overall map. **(E)** Local resolution map for the 3D reconstruction of the local structure. **(F)** Euler angle distribution in the final 3D reconstruction of local map.

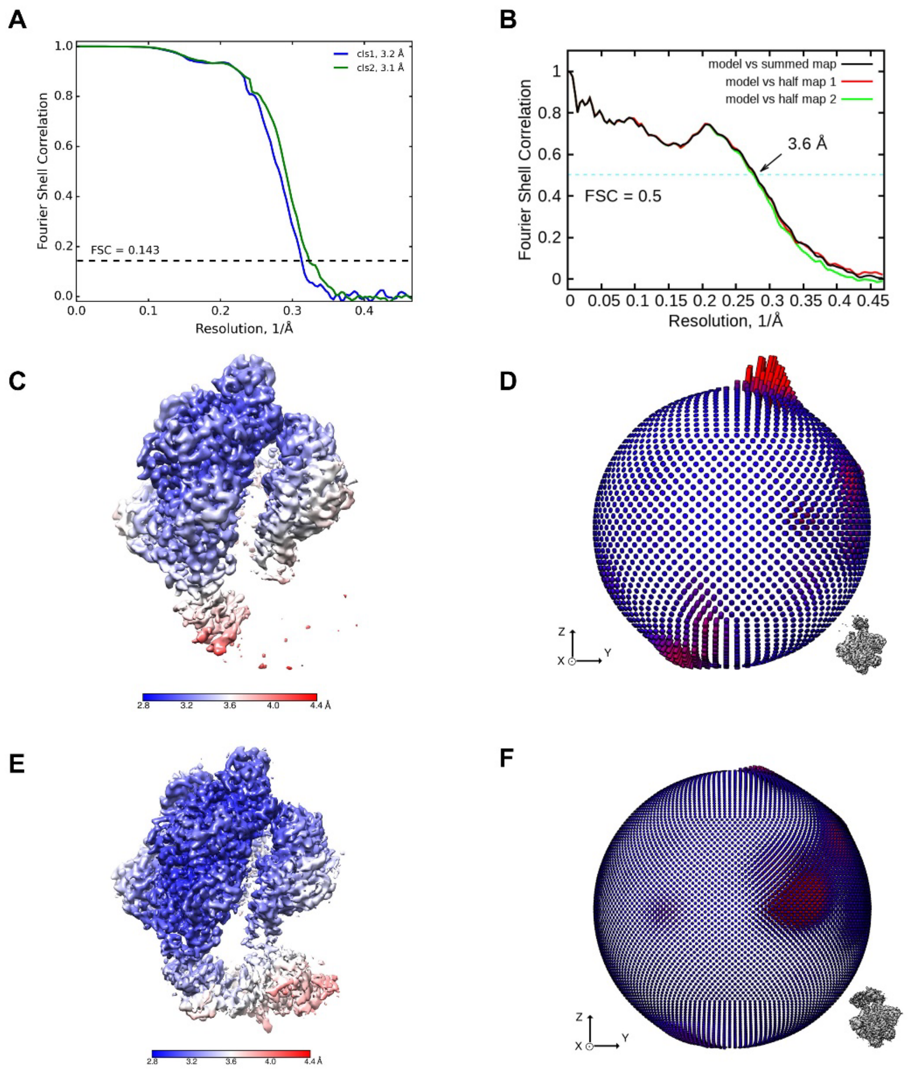

Fig. S2

Cryo-EM analysis of F8/A22/E4 complex in trimeric form.

(A) FSC curve. The resolution was estimated with the gold-standard Fourier shell correlation 0.143 criterion with high-resolution noise substitution. **(B)** FSC curve of the refined model versus the overall structure of class 2 that it is refined against (black); of the model refined against the first half map versus the same map (red); and of the model refined against the first half map versus the second half map (green). The small difference between the red and green curves indicates that the refinement of the atomic coordinates did not suffer from overfitting. **(C)** Local resolution map for the 3D reconstruction of the overall structure of class 1. **(D)** Euler angle distribution in the final 3D reconstruction of overall map of class 1. **(E)** Local resolution map for the 3D

reconstruction of the overall structure of class 2. **(F)** Euler angle distribution in the final 3D reconstruction of overall map of class 2.

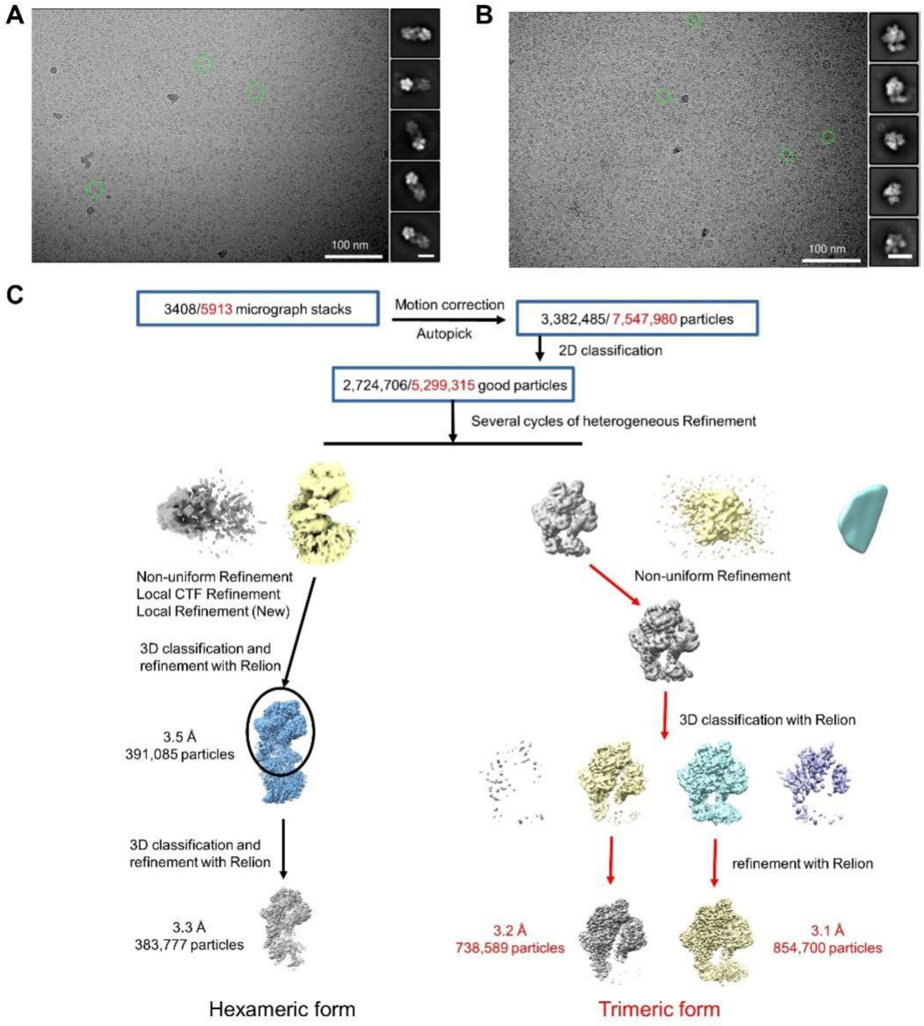

Fig. S3

Flowchart for cryo-EM data processing.

(A, B) Represent micrographs and 2D class averages. Scale bar of 2D, 10 nm.

(C) Flowchart for cryo-EM data processing. Please refer to the 'Data Processing' in Methods section for details.

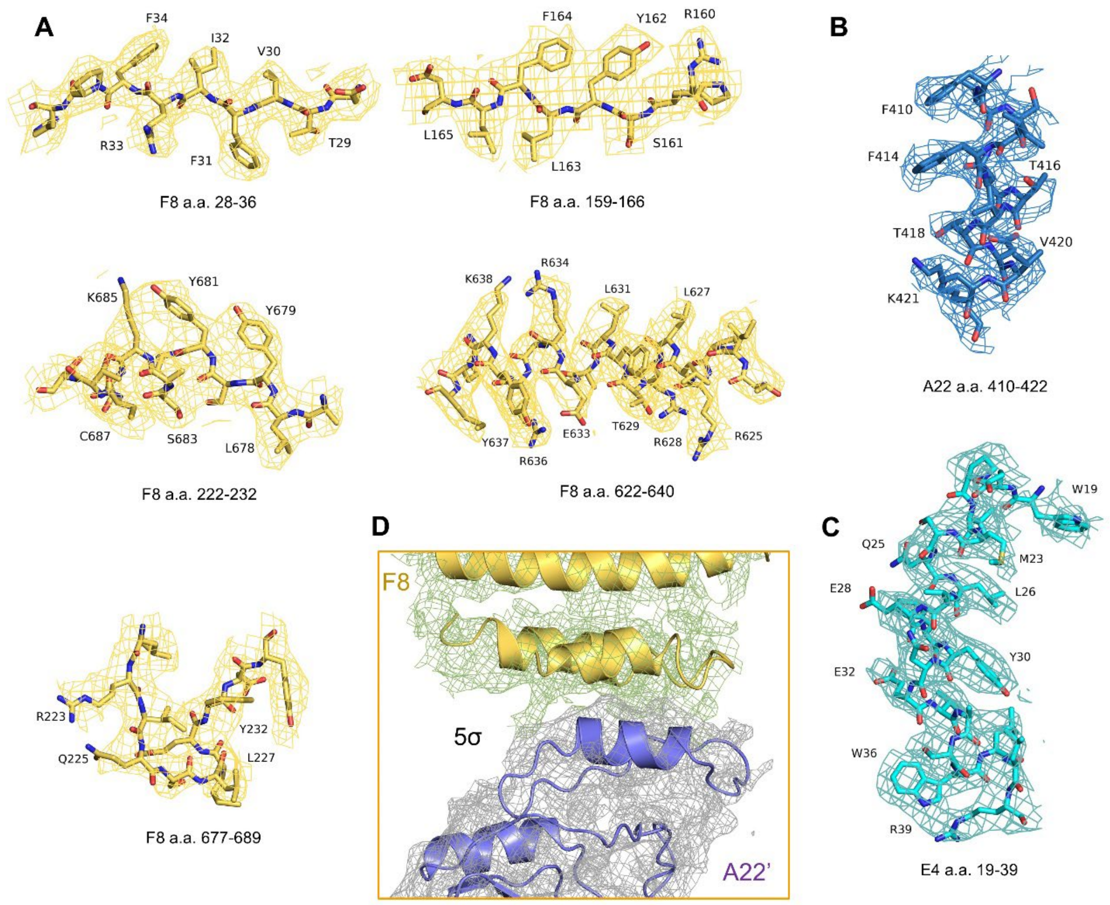

Fig. S4

Representative cryo-EM density maps of F8/A22/E4 complex in hexameric form.

(A-C) Cryo-EM density map of F8, A22 and E4 are shown at threshold of 7 σ. **D** Cryo-EM density map of interface between thumb domain of F8 and A22 is shown at threshold of 5 σ.

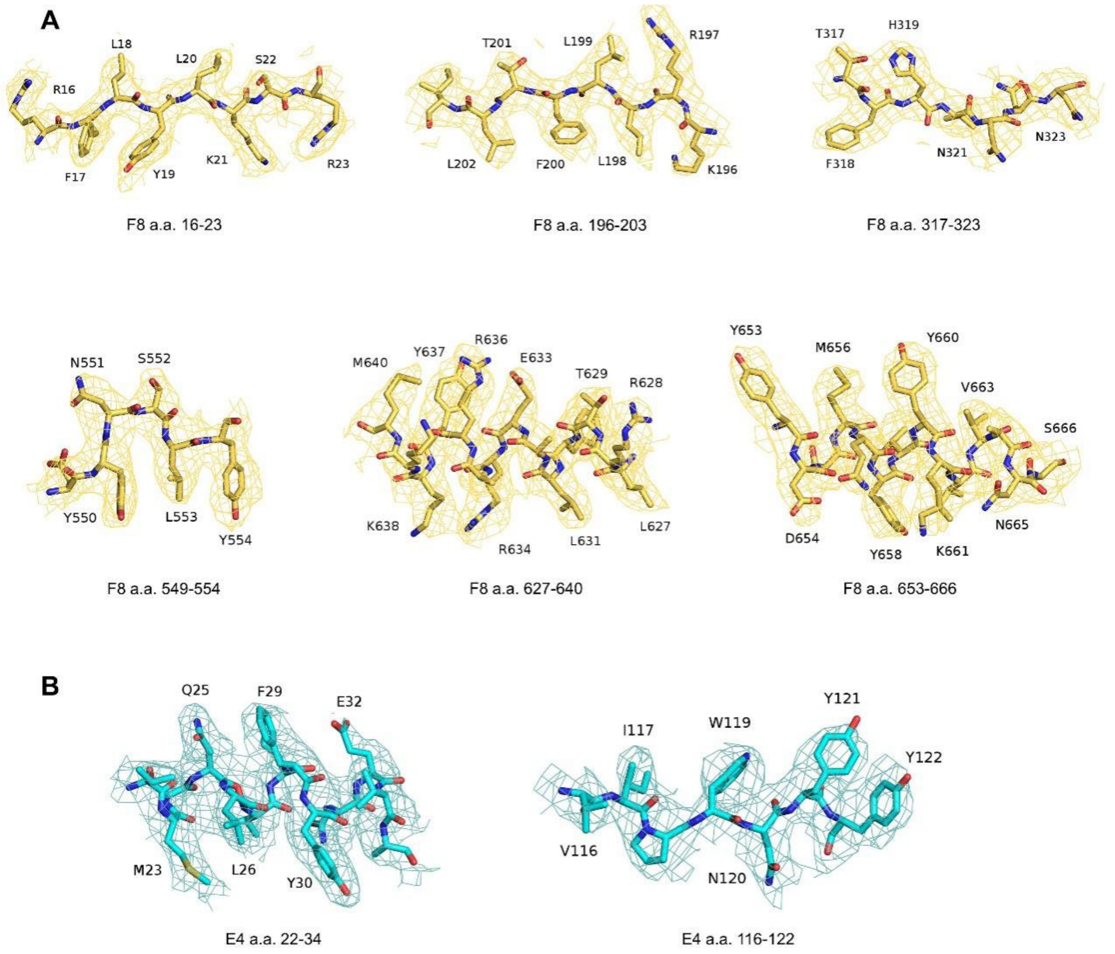

Fig. S5

Representative cryo-EM density maps of F8/A22/E4 complex in trimeric form.

(A) Cryo-EM density map of F8 are shown at threshold of 7 σ. **(B)** Cryo-EM density map of E4 are shown at threshold of 7 σ.

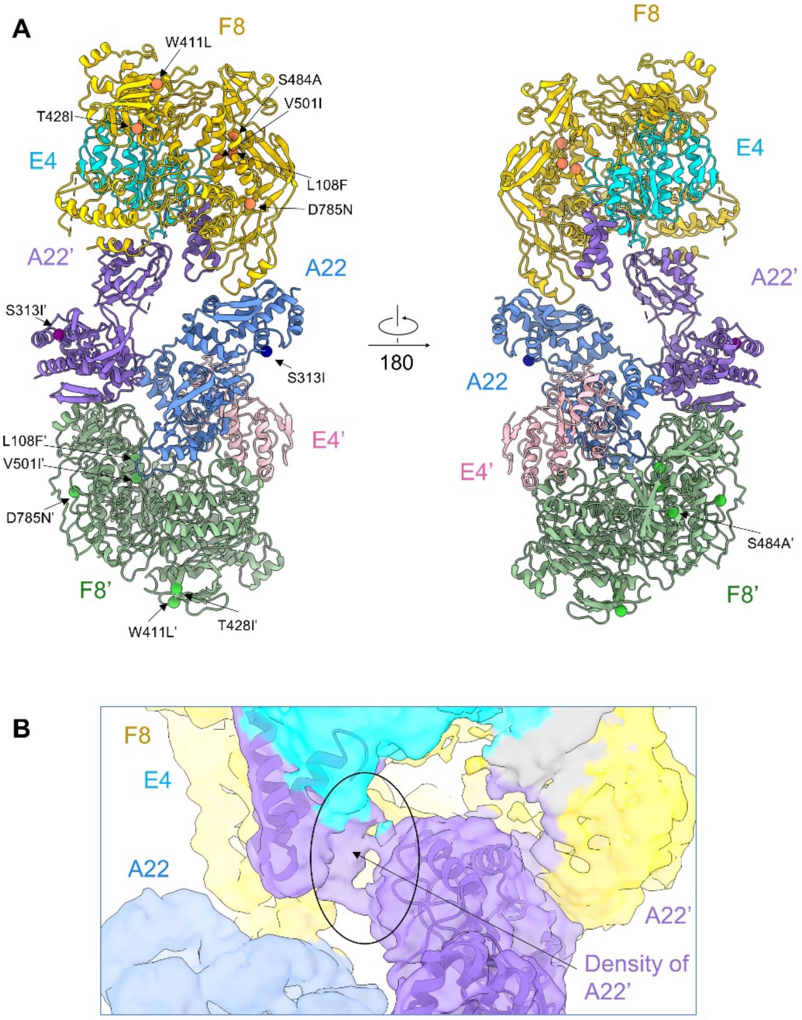

Fig. S6

The distribution of mutations of F8 and A22 from 2022 West African strain sub-lineages. (A) F8, A22 and E4 in protomer A are colored gold, blue and cyan, and the other protomer is colored green, purple and pink. Resides changed are styled sphere. Mutations on F8, F8', A22 and A22' are colored orange, green, blue, and purple, respectively. (B) Density of the linker between N-terminal helix and neck domain of A22'.

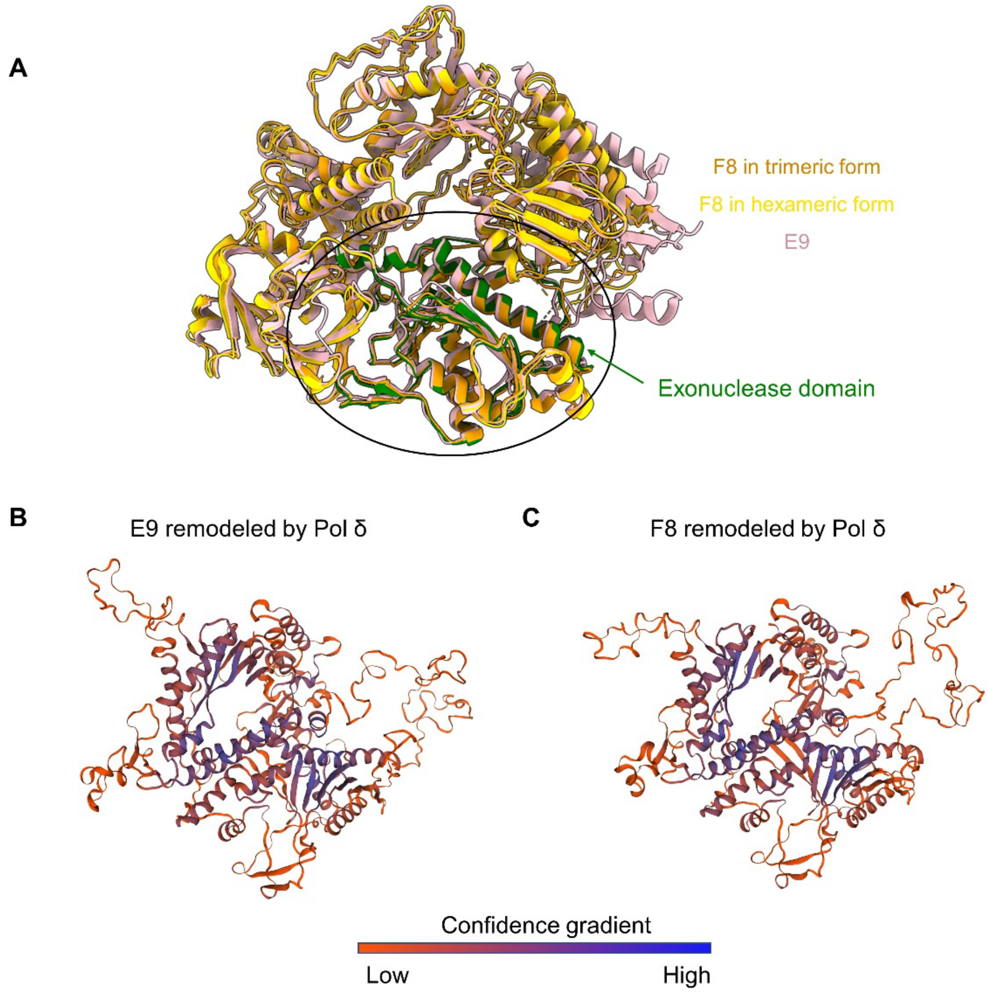

Fig. S7

Structural analysis of E9 in VACV and F8 in MPXV

(A) Structural comparison among F8 in trimeric form, in hexameric form and E9 alone (PDB ID:5N2E). F8 in trimeric form, in hexameric form and E9 alone are colored gold, orange and pink, respectively. Exonuclease domain of F8 in hexameric form are emphasized by green. (B, C) E9 in VACV (B) and F8 in MPXV (C) are remodeled using Pol δ (PBD ID: 3IAY) as template by SWISS-MODEL. Models are colored by confidence gradient.

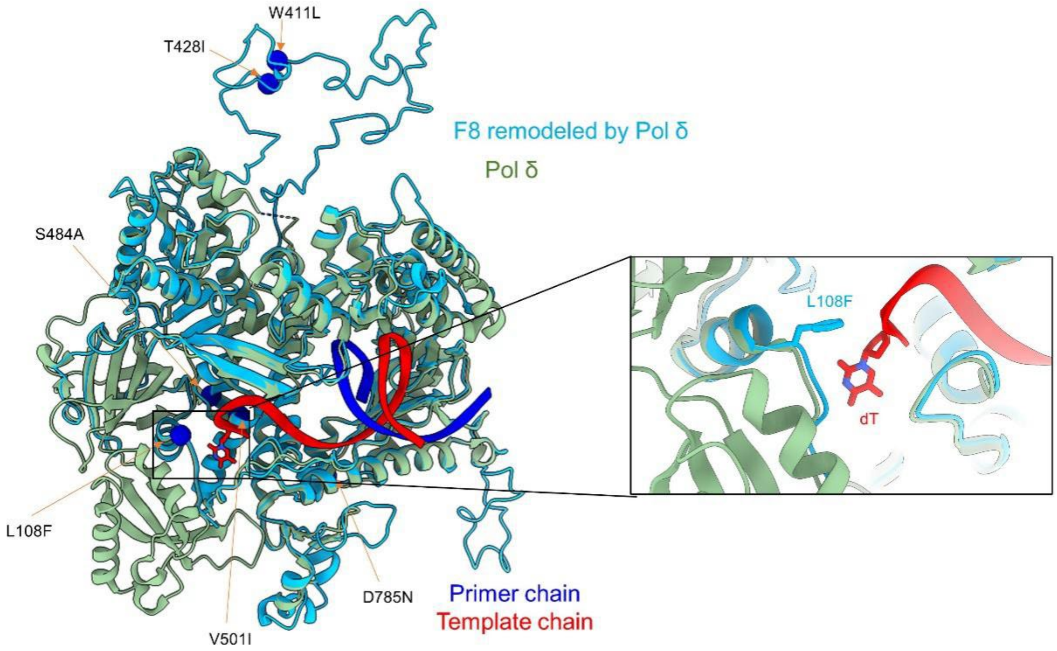

Fig. S8

The distribution of mutations of F8 from 2022 West African strain sub-lineages in F8 at close conformation.

Structural comparison between F8 at close conformation and Pol δ in complex with DNA (PBD ID: 3IAY) shows L108F may strengthen hydrophobic interaction with base of dT in DNA' template chain. F8 at close conformation and Pol δ are colored cyan and green, respectively. Primer chain and template chain of DNA are colored blue and red. Resides changed are styled sphere. F108 and dT are styled sticks.

Table S1

Cryo-EM data collection, refinement and validation statistics

**Data collection**		
EM equipment	Titan Krios (Thermo Fisher Scientific)	
Voltage (kV)	300	
Detector	Gatan K3 Summit	
Energy filter	Gatan GIF Quantum, 20 eV slit	
Pixel size (Å)	1.095	1.072
Electron dose (e-/Å2)	50	
Defocus range (µm)	-1.4 ~ -1.8	
Sample	F8-E4-A22 hexameric form	F8-E4-A22 trimeric form
Number of collected micrographs	3,408	5,913
**3D Reconstruction**		
Software	Relion/cryoSPARC	
Number of used particles (Overall)	391,085	854,700
Resolution (Å)	3.5	3.1
FSC threshold for resolution determination	0.143	0.143
Symmetry	C1	
Map sharpening B-factor (Å^2)	-90	
**Refinement**		
Software	Phenix	
Model composition		
Protein residues	3,034	1,467
Side chains assigned	3,032	1,276
CC-volume	0.46	0.78
CC-mask	0.42	0.80
B factors (Å^2)	4.40-140.79	17.72-192.86
R.m.s deviations		
Bonds length (Å)	0.010	0.005
Bonds Angle (°)	1.307	0.720
MolProbitiy score	3.61	2.91
Ramachandran plot statistics (%)		
Preferred	86.58	90.11
Allowed	11.66	9.82
Outlier	1.77	0.07
